# Neoadjuvant radiotherapy plus single-incision thoracoscopic surgery in the treatment of type B3 thymomas

**DOI:** 10.3389/fonc.2023.1094974

**Published:** 2023-03-23

**Authors:** Wenshan Li, Yimin Wu, Lijian Huang, Ying Chai

**Affiliations:** Department of Thoracic Surgery, Second Affiliated Hospital, School of Medicine, Zhejiang University, Hangzhou, China

**Keywords:** type B3 thymomas, neoadjuvant radiotherapy, single-incision thoracoscopy surgery, minimally invasive, myasthenia gravis

## Abstract

**Background:**

Although minimally invasive surgery is the standard treatment for thymomas, minimally invasive thymectomy is difficult for patients with type B3 thymomas, especially for giant or aggressive lesions. These tumors are frequently treated with radical radiation therapy or surgery plus adjuvant radiotherapy. Few studies, however, have tested the efficacy of neoadjuvant radiotherapy prior to thoracoscopic surgery.

**Methods:**

Patients with type B3 thymomas >5 cm or with infiltrates into vital organs on CT-guided puncture biopsy who underwent neoadjuvant radiotherapy followed by single-incision minimally invasive thymectomy from March 2016 to July 2020 were retrospectively evaluated. Reduction ratios, TNM stage changes according to WHO stage criteria, resectability, long-term survival, and the response in terms of RECIST v1.1 criteria achieved by preoperative RT were analyzed.

**Results:**

The 11 patients who underwent neoadjuvant radiotherapy plus minimally invasive thymectomy included five men and six women, of mean age 49.5 years. Four patients had myasthenia gravis. Neoadjuvant radiotherapy consisted of 50 Gy in 25 fractions, with all patients showing varying degrees of lesion reduction after radiotherapy. Surgery was performed about 1 month after neoadjuvant radiotherapy, with none of these patients having severe radiation pneumonitis. All patients underwent radical resection of the tumor and adjacent tissue, with none experiencing tumor seeding or rupture during surgery. The median postoperative hospital stay was 3 days (range: 2–6 days) and the frequency of additional regular analgesics (including those for wound pain and neuralgia) was 2.5 times per person. On follow-up, one patient experienced pleural metastasis and one experienced pulmonary metastasis, with the other nine patients showing no evidence of tumor recurrence.

**Conclusion:**

Neoadjuvant radiotherapy followed by minimally invasive surgery was a safe and efficacious procedure for the treatment of type B3 thymomas, with less postoperative pain and faster recovery. This strategy, of tumor shrinkage prior to surgery, may make possible the easier removal of type B3 thymomas by single-incision thoracoscopy.

## Introduction

Thymomas are intrathoracic neoplasms of the thymus, with an age standardized rate of 0.15 to 0.19 per 100,000 person-years (1). Although rare, thymomas remain the most common primary malignancy in the mediastinum (1). The malignant potential of thymomas varies widely. Most thymomas can be treated by radical surgical resection, with most of these tumors currently removed by minimally invasive surgical methods (2). Giant and invasive thymomas, especially type B3 thymomas which size >5 cm or with infiltrates into vital organs with the stage of T1-3N0M0 based on the Masaoka classification and the eighth edition of the Union for International Cancer Control/American Joint Committee on Cancer TNM stage classification, however, are generally resected *via* multi-incision thoracoscopy or an open approach.

The core principles of thymectomy are visualization of the phrenic nerve and left innominate vein, as well as complete phrenic to phrenic resection. Many type B3 thymomas, however, are difficult to expose and resect by single incision thoracoscopy. Neoadjuvant radiotherapy may reduce the size of these lesions significantly, allowing subsequent surgical resection. The present study describes the experience of our center with preoperative adjuvant radiotherapy followed by minimally invasive, single incision thoracoscopic surgery in the treatment resection of type B3 thymomas.

## Methods

The medical records of all patients with type B3 thymomas who underwent preoperative adjuvant radiotherapy, followed by R0 resection using single incision thoracoscopy from March 2016 to July 2020 were retrospectively reviewed. All patients with anterior mediastinal tumors were evaluated by CT-guided puncture biopsy. Patients with tumors having features highly suggestive of thymoma, with signs of invasion of surrounding structures or a diameter >5 cm were further evaluated. Those with tumors histologically confirmed to be type B3 thymomas were treated with the neoadjuvant radiotherapy, consisting of 50 Gy in 25 fractions of 2 Gy each. We have used preoperative XRT in the doses of 50Gy/25F in expectation of increasing the rate of complete resection, and avoiding postoperative radiotherapy, and the operation was technically difficult with a higher radiation doses like 60Gy.

Surgery was usually performed around 1 month after the end of radiotherapy. The reason why we choose to operate 1 months after XRT is to avoiding making resection difficult because of XRT induced edema in shorter time and induced adhesion in longer time in operative area. All patients were re-tested prior to surgery. Testing included measurements of pulmonary function, CT, ECG and basic blood tests, with specific parameters, such as Ach-receptor and anti-MuSK and anti-Titin antibodies, also measured in patients with myasthenia gravis (MG).

**Figure 1:**
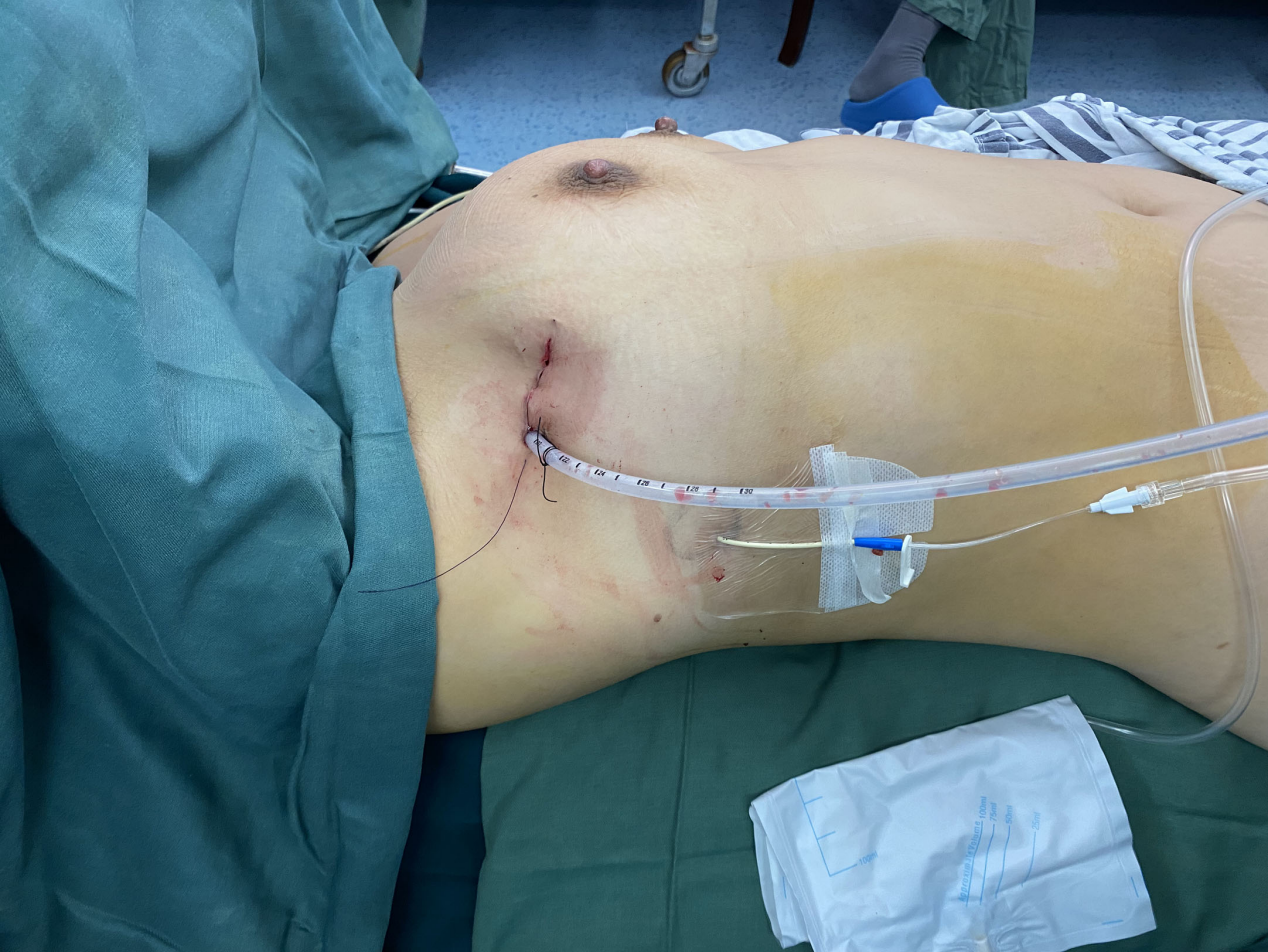
The indwelling of postoperative drainage tube. Two drainage tubes ensured adequate postoperative drainage. The 22F tube was removed within 24-h in order to reduced perioperative pain, and the CVC tube was removed at patient discharge.

The surgical approach was usually from the right thoracic cavity, except when the lesion was located predominantly on the left side. Patients were anesthetized using a double-lumen tube for split-lung ventilation and placed unilateral-side up at an angle of 30°. Usually, a 5–6 cm incision was created at the anterior axillary line in the fourth intercostal space. The dissection generally started inferiorly after inspection of the thymus gland and the thymoma, with initial dissociation along the right phrenic nerve, followed by exposure of the left innominate vein until the superior horns were identified. The thymus vein was usually severed when the tumor was sufficiently freed. Lesions were removed from the cavity in a surgical retrieval bag. A drainage tube, usually a 22F tube, was inserted into the incision and a CVC tube was inserted into the sixth intercostal space at the axillary midline (**Figure 1**). The 22F tube was removed within 24-h, and the CVC tube was removed at patient discharge. The surgical approach was similar with those who do not undergo XRT, which were generally resected *via* multi-incision or bilateral thoracoscopy, and the 22F tube was removed later, generally 2-3 days.

The diagnoses of all resected lesions, surgical margins, and Masaoka stage were further confirmed by histological examination. Follow-up generally consisted of semi-annual CT examinations. Patients with MG were administered acetylcholinesterase inhibitors throughout the treatment phase.

## Results

**Figure 2:**
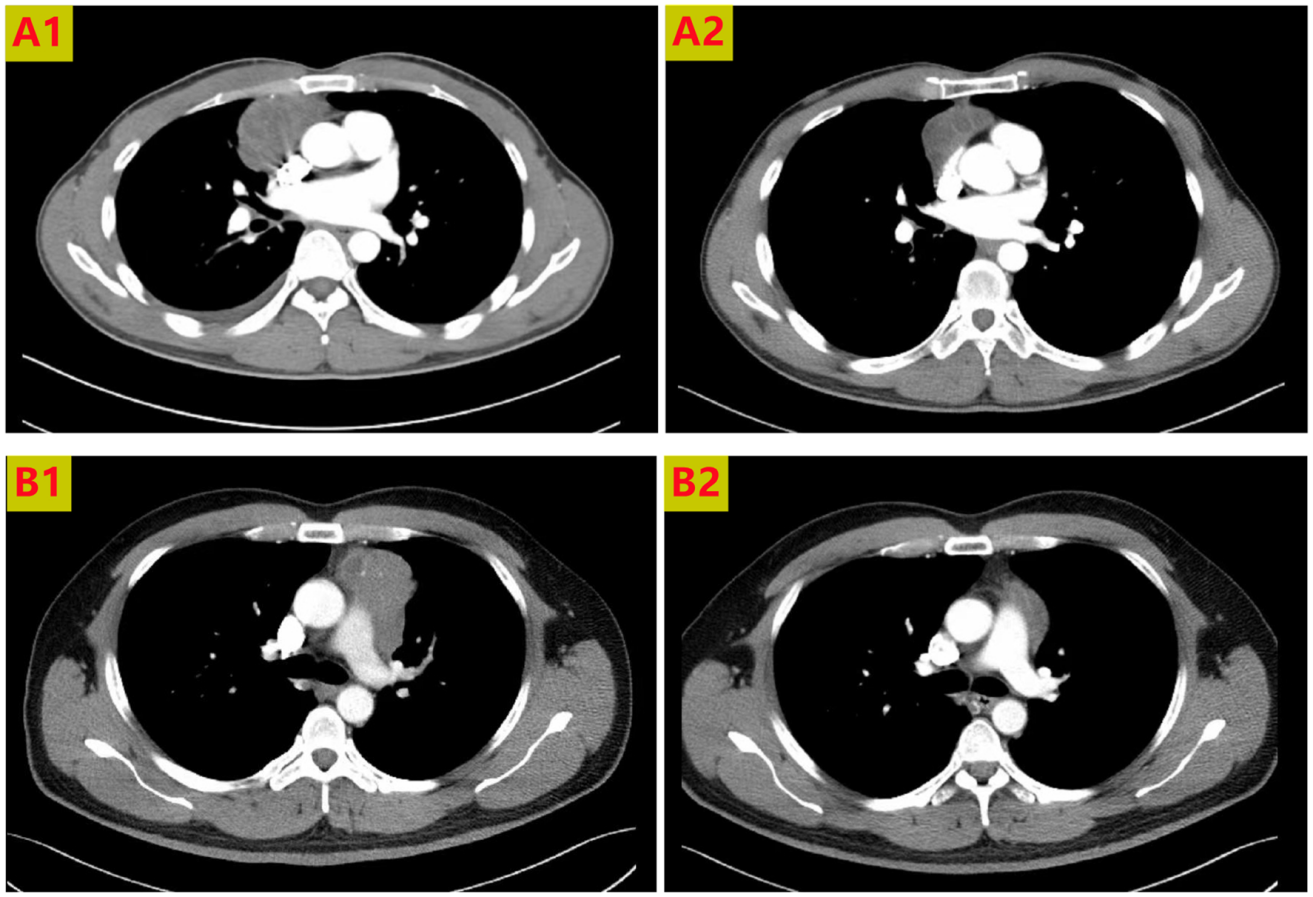
Chest CT scans of 2 patients receiving neoadjuvant radiotherapy. The anterior mediastinal lesion shrank more than two-fold in volume. Thoracic CT scans showing tumors before treatment **(A1, B1)**, and partial response to radiotherapy **(A2, B2)**.

The current study enrolled 11 patients, five men and six women, of mean age 49.5 years (49.5 ± 7.6). Four patients had MG. All patients showed varying degrees of lesion reduction after radiotherapy (**Figure 2**). However, three patients required extended resection including the pericardium or lung wedge resection.

All patients underwent single-incision minimally invasive R0 resection without macroscopic infiltration of the thymic bed. Moreover, microscopic examination of tumor margins showed that all were negative. None of these patients required emergency conversion to open thoracotomy or blood transfusion. One patient experienced postoperative pneumothorax, whereas none of the other 10 experienced surgical complications, such as massive bleeding or nerve damage.

The median postoperative hospital stay of all 11 patients was 3 days (range 2–6 days). The median postoperative hospital stay was 3 days (range: 2–6 days) *vs* 4.5 days (range: 2–9 days) compare with the conventional treatment patients (giant or aggressive lesions treated with surgery plus adjuvant radiotherapy) during the same period, and the frequency of additional regular analgesics (including those for wound pain and neuralgia) was prescribed significantly lower than that in the conventional treatment patients during postoperative hospitalization (2.5 *vs* 4.2). The diagnoses of all resected thymomas, surgical margins, and Masaoka stage were confirmed by histological examination. Of these 11 patients, two had stage I, five had stage II and four had stage III thymomas. The characteristics of all patients and their surgical outcomes are summarized in **Table 1**. The characteristics of the response in terms of RECIST v1.1 criteria achieved by radiotherapy are summarized in **Table 2**.

**Table 1:** Characteristics and Surgical outcomes of the patients.

Demographics	Perioperative outcomes
Age median [range]	49.5Y (31-68)
Male/Female	5/6
MG	4
Postoperative hospital stay, median [range]	3D (2-6)
Surgical complications	1 (pneumothorax)
Follow-up time median[range]	34M (19–67)
Recurrence	2 (1 pleura, 1 pulmonary)
Masaoka stage	
I	2
II	5
III	4

**Table 2:** The response in terms of RECIST v1.1 criteria achieved by radiotherapy.

No.	Size	TNM stage (at diagnosis)	Size (after XRT)	TNM stage (after XRT)	Response
Case 1	7.6	T2N0M0	4.9	T2N0M0	PR
Case 2	6.8	T1bN0M0	3.8	T1aN0M0	PR
Case 3	5.9	T3N0M0	3.1	T2N0M0	PR
Case 4	4.9	T2N0M0	3.0	T2N0M0	PR
Case 5	5.2	T2N0M0	3.2	T2N0M0	PR
Case 6	5.1	T3N0M0	3.8	T3N0M0	SD
Case 7	7.7	T3N0M0	4.1	T3N0M0	PR
Case 8	4.8	T3N0M0	2.9	T2N0M0	PR
Case 9	8.7	T3N0M0	4.2	T3N0M0	PR
Case 10	6.3	T3N0M0	4.0	T3N0M0	PR
Case 11	5.5	T1aN0M0	3.5	T1aN0M0	PR

The median follow-up time was 34 months (range 19–67 months). One patient experienced a recurrence in the pleura after 1.7 years and another experienced a pulmonary recurrence after 4.1 years, with the latter receiving further chemoradiotherapy. In addition, two of the four patients with MG showed improvements after surgery.

## Comment

Primary tumors in the anterior mediastinum account for half of all mediastinal masses, with thymomas being the most common type of primary anterior mediastinal tumors. Their invasiveness, however, varies widely. Most thymomas can be removed surgically through minimally invasive access by thoracoscopic or robotic approaches (3). Single incision thoracoscopic surgery provides minimally invasive access and has the advantages of less pain, shorter hospital stay, superior cosmetic outcomes, and faster postoperative rehabilitation. The use of single incision thoracoscopic surgery, however, is more limited in patients with type B3 thymomas, especially giant invasive lesions. Although our center has had extensive experience with single-incision thoracoscopic treatment of benign or less aggressive thymomas, additional incisions or conversion to open thoracotomy may be required for giant invasive thymomas, allowing only R1 resection.

Radiotherapy is widely used to treat type B3 thymomas, but mostly for postoperative adjuvant treatment (4, 5). We accidentally found that an aggressive type B3 thymoma lesion shrank significantly after radiotherapy, making radical treatment *via* single-incision thoracoscopy smoothly. Although primary surgery may be sufficient for smaller lesions (<5 cm) that did not invade vital structures, Neoadjuvant radiotherapy followed by single-incision thoracoscopy may provide better outcomes in type B3 thymomas >5 cm in diameter and those infiltrating vital organs. Preoperative pathology can be assessed by CT-guided biopsy, without bleeding or infection.

Although robotic-assisted thoracoscopic surgery is a good approach, it is rarely used in our center due to its excessive costs. Robotic approaches, however, have the advantages of greater surgical field exposure. Great care must be taken to avoid injuries to vascular structures and nerves, with bleeding, especially from the left innominate vein, being the most serious surgical complication. The advantages of the right-sided approach include exposure of the visual field of the left innominate vein, allowing gradual separation from the proximal to the distal end, thus reducing the likelihood of intraoperative bleeding. Surgical access from the right side may therefore be preferable, although left-sided access or multi-port surgery may be superior in identifying and processing the region of the upper thymic horn and the thymic tissue in the aortic-pulmonary window.

Surgical treatment of type B3 thymomas in patients with MG is especially challenging. Perioperative myasthenic crises can have serious consequences, such as airway obstruction and aspiration. None of the patients in the present study, study, experienced these complications. Minimally invasive surgical techniques, use of regional analgesia, and administration of acetylcholinesterase inhibitors during the perioperative period are therefore essential (6). In addition, preoperative immunoglobulin therapy is recommended if allowed. Complete resection of the tumor, along with surrounding fatty and mediastinal tissue, is of paramount importance and is associated with good prognosis.

Postoperative drainage tubes were inserted into all patients included in this study. The 22F tubes were removed within 24 hours, and the CVC tube ensured adequate drainage of postoperative fluid with less pain and shorter hospital stay. Due to the possibility of rapid recovery, CVC chest tubes alone were inserted into some patients, without significant fluid pneumothorax being present.

All 11 patients in this series underwent complete resection, with none receiving further chemoradiotherapy. Unfortunately, two patients experienced postoperative recurrence. Because this was a retrospective, small sample, single-center study, recurrence rates could not be compared with other postoperative adjuvant treatment options. Thymomas and most thymic carcinomas have been reported to lack targetable mutations and to have extremely low tumor mutational burden, but usually have a programmed death-ligand 1 (PD-L1) high phenotype (7), indicating that immunotherapy has great potential to improve patient prognosis. Currently, we are testing a preoperative regimen, consisting of immunotherapy combined with radiotherapy, which has been found to significantly expand treatment indications and reduce recurrence rates. However, further validation is required to determine the likelihood and magnitude of potential benefits.

In order to get better survival, various modalities of preoperative chemotherapy such as adriamycin, cisplatin, vincristine or cyclophosphamide, have been adapted as an induction therapy for the management of stage II-IV thymomas. Based on available data, the most popular and active regimens are cisplatin-anthracycline (CAP or ADOC) or cisplatin-etoposide combinations, which showed response rates above 50% in most of the series, whatever in front-line therapy or in case of relapsing tumours (8). However, these therapeutic regimens have not reached a satisfactory result (9–11). The study conducted by Ma et al. found that different front-line chemotherapy regimens of cisplatin, doxorubicin, and cyclophosphamide (CAP); cisplatin and etoposide (EP); or cisplatin and paclitaxel (TP) may provide similar long-term PFS and OS in patients with advanced thymoma (12). Fornasiero et al. assessed 32 patients with stage III or IV thymoma who received the first‐line cisplatin, doxorubicin, vincristine, and cyclophosphamide (ADOC) regimen, and the result showed an ORR to the ADOC regimen of 91.8%, but the median OS for patients undergoing this regimen was only 15 months (13). In another study, Kim et al. conducted a phase II study using a multidisciplinary approach of induction chemotherapy followed by surgical resection, radiation therapy, and consolidation chemotherapy for patients with unresectable malignant thymoma. Twenty‐two patients received the CAP regimen with prednisone. The ORR was 77% after induction chemotherapy, and the 5-year PFS rates was only 77% (14). Besides, postoperative radiotherapy (PORT) has been widely used as an adjuvant therapy to improve survival rates and reduce recurrence rates, as thymomas are sensitive to radiation therapy. From seven retrospective series analysis of 1724 patients with sole primary thymomas, there is no difference between PORT and Masaoka stage II disease in overall survival (HR 1.45, 95% CI 0.83-2.55). However, improved OS was observed with the addition of PORT in the discrete pooled analysis of stage III to IV (HR 0.63, 95% CI 0.40-0.99) (15). This is the main reason why we chose radiotherapy as preoperative adjuvant therapy.

Of course, our study has limitations. The disease is so rare that it is very difficult to obtain a large series of patients. In fact, we have tried to collect the date of patients who received radical operation without neoadjuvant radiotherapy in order to set a control group at the same time. However, we found that the tumor staging of patients in control group is obviously early than experimental group. So we have to cancel this group. That’s lead to a lack of a systematic statistical comparison.

All lesions showed promising shrinkage after radiotherapy, making it easier to remove the tumor *via* a single incision. Although none of these patients experienced severe radiation pneumonitis, radiotherapy associated pleural adhesions increased operation complexity. Resection should be performed by thoracic surgeons experienced in both extended open and minimally invasive surgery. Although preoperative radiotherapy followed by R0 resection may not be suitable for all patients with enlarged and invasive lesions, this study showed that this strategy was feasible in the treatment of type B3 thymomas.

## Data availability statement

The original contributions presented in the study are included in the article/supplementary material. Further inquiries can be directed to the corresponding authors.

## Ethics statement

The studies involving human participants were reviewed and approved by The Ethics Review Board of the Second Affiliated Hospital of the Zhejiang University School of Medicine (Zhejiang, China). The patients/participants provided their written informed consent to participate in this study.

## Author contributions

Dr WL drafted the manuscript and statistics assessment. Dr LH is involved in date collection and patient follow-up. Dr YW was responsible for checking the imaging tests and assessing the response in tumor. Dr YC is involved in collect the date and modifying the manuscript. All authors contributed to the article and approved the submitted version.
